# Quality of Life Assessment in Trials of Revascularization for Chronic Stable Angina: Insights from ORBITA and the Implications of Blinding

**DOI:** 10.1007/s10557-021-07198-8

**Published:** 2021-08-21

**Authors:** Alexandra N Nowbar, Darrel P Francis, Rasha K Al-Lamee

**Affiliations:** 1 National Heart and Lung Institute, Imperial College London, W12 0HS, London, UK; 2 Imperial College Healthcare NHS Trust, Hammersmith Hospital, NHLI - Cardiovascular Science, B block South, 2nd floor, Du Cane Road W12 ONN, London, UK

**Keywords:** Stable angina, Percutaneous coronary intervention, Coronary artery disease, Quality of life, Seattle Angina Questionnaire, EuroQoL EQ-5D-5L

## Abstract

The main aims of therapy in chronic stable angina are to reduce the risk of myocardial infarction and death and improve symptoms and quality of life (QoL). Unblinded trials have shown that revascularization does not reduce the risk of myocardial infarction or death but does appear to improve symptoms. However, symptoms are susceptible to the placebo effect which can bias therapies to appear more effective than they are. To assess the true physical impact of a treatment on symptoms, placebo-controlled trials with patients and medical and research teams blinded to treatment allocation are necessary. Symptoms and QoL can be reported directly by the patient or indirectly by the physician. Patient-reported outcome measures in angina trials can include angina frequency, frequency of nitrate use, exercise capacity, and questionnaires such as the Seattle Angina Questionnaire (SAQ) and the generic EuroQOL-5D-5L (EQ-5D-5L) QoL questionnaire. Physician-assessed outcome measures include Canadian Cardiovascular Society Class. The Objective Randomised Blinded Investigation with Optimal Medical Therapy of Angioplasty in Stable Angina (ORBITA) trial was the first blinded placebo-controlled study investigating the role of percutaneous coronary intervention (PCI) in chronic stable angina. The trial showed a smaller than expected and not statistically significant effect of placebo-controlled PCI on the primary endpoint of change in exercise time at 6 weeks follow-up in single-vessel coronary artery disease. There was also no significant placebo-controlled treatment effect of PCI for the prespecified secondary endpoints of SAQ or EQ-5D-5L, although PCI did result in 20% more patients becoming free from angina than placebo in a non-prespecified secondary analysis. ORBITA has demonstrated the need for symptomatic and QoL effects of PCI to be studied using placebo control. Here, we describe ways of measuring QoL, the impact of the unblinded and blinded trials to date, what we have learned from ORBITA, and what is next for this common and complex condition.

## Background

Chronic stable angina is estimated to affect over 9 million adults in the USA (a prevalence of 3.6%) [1]. It has a considerable impact on quality of life (QoL) [2].

The aims of therapy for angina are two-fold: (i) to reduce cardiovascular risk and (ii) to relieve symptoms and improve QoL. The latter is particularly important in light of the results of the International Study of Comparative Health Effectiveness with Medical and Invasive Approaches (ISCHEMIA) [3] and Clinical Outcomes Utilizing Revascularization and Aggressive Drug Evaluation (COURAGE) [4] trials which showed little or no prognostic advantage of revascularization, even in patients with moderate-to-severe burden of ischemia, resulting in the primary remit of revascularization to improve QoL in patients with stable symptoms, preserved left ventricular function, and no significant left main stem disease.

This paper examines QoL assessment in angina trials, the QoL results of trials of percutaneous coronary intervention (PCI) and coronary artery bypass grafting (CABG), and the impact of blinding.

## Endpoints in Trials of Chronic Stable Angina

The reporting of symptoms and QoL can vary between patients and even within patients at different timepoints. Inter- and intra-patient variability can be affected by mood; comorbidities; and situational, social, and economic circumstances. The way in which we tolerate medical conditions and pain varies and is influenced by physical, psychological, and social factors. Qualitative reporting with description of the nature, impact, and change in symptoms and QoL is also frequently used in clinical practice. Quantitative tools have been developed to assess symptoms and QoL. These allow reproducible and standardized assessment through application of scoring systems to qualitative data.

Symptoms and QOL can be reported by the patient or assessed by the physician. Physician-assessed and patient-reported symptoms may differ [5]. The patient’s self-assessment is the most direct measure in determining the utility of a therapy. QoL assessment tools can be disease-specific or generic.

Physician assessment of symptoms and QoL has value as it incorporates clinical judgement into the evaluation. Physician-assessed symptom evaluation includes the Canadian Cardiovascular Society (CCS) class grading of angina symptoms.

Patient-reported assessment frequently incorporates questionnaires such as the Seattle Angina Questionnaire (SAQ) and EuroQol-5 (EQ-5D-5L). These allow numerical quantitative assessment of symptoms facilitating comparison of symptoms over time in individual patients, between patients, and between groups. They are used to evaluate symptom burden and treatment effects and deliver some consistency.

Table 1 summarizes commonly used angina endpoints.

**Table 1:** Commonly used endpoints in angina trials

Measure	Physician-assessed or patient-reported	Description	Example trial	Limitation
CCS class	Physician-assessed	Functional capacity (I, II, III, or IV)	RITA-1 [6]	Low relevance to patient due to being physician-assessed, low power due to being only a 4-point scale
SAQ	Patient-reported	Five domains (physical limitation, angina stability, angina frequency, treatment satisfaction, quality of life)	COURAGE [4]	Recall bias, affected by patient’s level of exertion
Sublingual nitrate consumption	Patient-reported	Diary of patients’ self-report of use of nitrate	TERISA [7]	Affected by patient’s level of exertion, sublingual nitrate use is not universal
Angina frequency	Patient-reported	Diary of patients’ self-report of angina episodes	ERICA [8]	Affected by patient’s level of exertion
SF-36	Patient-reported	Five domains (general health, limitations of activities, physical health, emotional health, and social activities)	QUART [9]	Generic health-related quality of life
EQ-5D-5L	Patient-reported	5 domains (mobility, self-care, usual activities, pain/discomfort and anxiety/depression)	ISCHEMIA [3]	Generic health-related quality of life, ceiling effects
MacNew	Patient-reported	Three domains (physical, psychological, and social)	EXIT [10]	Heart-specific but not angina-specific
Exercise testing	N/A	Can include time to ST depression, peak oxygen uptake, exercise time	ORBITA [11]	Influenced by comorbidities such as respiratory disease or musculoskeletal conditions

*EQ-5D* 5-dimension EuroQoL, *QoL* quality of life, *SAQ* Seattle Angina Questionnaire, *SF-36* 36 item short form survey

Angina diaries are a useful longitudinal method of recording angina episodes and sublingual nitroglycerin use. Patient recorded diaries were used to track the effect of ranolazine on angina in women with ischemic heart disease [12] and in a comparison study of bisoprolol and atenolol [13]. Angina diaries are the gold standard for assessing angina frequency as episodes are recorded contemporaneously and not based on patient recall. They have been used to validate SAQ angina frequency [14, 15].

The Angina Pectoris Quality of Life Questionnaire (APQLQ) is a disease-specific QoL questionnaire. It was validated against exercise time and time to onset of pain with correlation coefficients of approximately -0.4 [16]. However, it has been described as requiring further work to assess its responsiveness to change and its test-retest reliability [17].

The SAQ [18] is also disease-specific and allows quantitative assessment across 5 domains and has been validated across a broad range of patients. The questions explore common activities of daily life including walking, gardening, and carrying groceries. It asks patients to report their angina symptoms over the preceding 4 weeks and whether medications were taken to alleviate symptoms. It also explores their satisfaction with treatment and how satisfied they would be if their symptoms remained the same for the rest of their lives. It is measured on a scale of 0 to 100, with higher scores representing more favorable angina status. Disease-specific instruments are useful when the aim of a trial is to determine the on-target effects like improvements in pain and physical limitation.

The EQ-5D-5L (5 level version of EuroQol-5 dimensions) [19] and the Nottingham Health Profile (NHP) are generic QoL questionnaires that have been used to assess QoL in angina trials [20]. Unlike EQ-5D-5L, NHP also covers sleep and energy dimensions which are particularly affected by angina and therefore important for assessing a therapy. However, the main advantage of EQ-5D-5L is that it allows cost-utility analysis through calculation of quality-adjusted life years (QALYs). A key limitation, though, is that many patients score at the top of all the EQ-5D-5L domains [21]. This distribution may make it difficult to track changes in angina if baseline quality of life is too “good.”

Generic questionnaires may be more useful than disease-specific ones for evaluating a therapy because they provide a fuller picture of a patient’s QoL [22]. For example, a therapy might dramatically reduce chest pain and decrease physical limitations but might cause intolerable headaches. Alternatively, a therapy might show no advantage in exercise time but reduce the need for additional medication which could improve the overall QoL. These subtleties would be captured by a generic QoL questionnaire but not by measures of angina frequency, sublingual nitrate use, or SAQ. This could make the difference between medical therapy and PCI or the difference between PCI and CABG.

While not a specific assessment of symptoms or QoL, exercise testing is often used to provide a numerical assessment of exercise capacity and exercise-induced symptoms. Cardiopulmonary exercise test (CPET) additionally allows measurements of gas exchange offering a more holistic evaluation of a patient’s cardiac, respiratory, and metabolic systems. This may provide a more objective real-time insight into a patient’s physiological and functional status than questionnaires. One limitation is that it can be influenced by non-cardiac comorbidities. Another limitation is that some patients who report angina in their everyday lives do not find the exercise test reproduces their symptoms.

No tool can guarantee to assess what is important for each individual patient. More questions and more frequent monitoring may help but can be an additional burden to patients, e.g., increasing anxiety, and make interpretation of the results more contentious.

## Bias-Resistant Endpoints

Bias can be introduced into a study before and after the intervention is delivered. Randomization reduces bias before the intervention, e.g., preventing clinicians favoring sicker patients for the intervention. Blinding minimizes bias after the intervention, e.g., preventing clinicians reporting better outcomes in patients who had the intervention.

Endpoints are sometimes colloquially described as “hard” (e.g., myocardial infarction or death) or “soft” (e.g., symptoms or QoL), but this misses the fundamental problem which is vulnerability to bias. In an unblinded trial, death is invulnerable to bias in ascertainment, but symptoms are extremely vulnerable. For this reason, in an unblinded trial, mortality is far more resistant to bias than symptoms. However, the origin of this distinction is not the nature of what is being measured, but rather, whether that measurement is susceptible to bias from knowing which arm the patient is in.

It is unwise to use “hard/soft” terminology when appraising clinical trials because it fundamentally misrepresents the endpoint as the problem, when in fact it is the unblinded process of measurement that causes it to be unreliable.

Endpoints such as angina and QoL are crucial and are not “soft.” It is measuring them unblinded that is “soft.” Pain is the epitome of a phenomenon that is vulnerable to bias by the placebo effect. Knowing whether one has received active therapy or placebo (e.g., as is normal in clinical practice or in an open-label trial) biases the reporting of endpoints like pain.

While the use of quantitative tools may improve the accuracy and reproducibility of symptom and QoL assessment and facilitate analysis, they cannot remove bias. In clinical practice, we are aware that different patients report symptoms and QoL in different ways. We are sometimes less aware of the impact of our own bias or preconceived beliefs on the assessment of symptoms and QOL. For example, we may be more likely to report that a patient’s symptoms have improved when we know they have received therapy that we think works. In clinical trials, this biased interpretation can only be addressed by trial design.

Randomization allows researchers to measure the impact of an intervention on patients through minimizing biased allocation. Placebo-control equalizes patient and staff expectations in the different arms of a trial.

The impact of the placebo effect can be eliminated by subtracting the effect in the placebo arm from that in the active arm.

Bias-resistant placebo-controlled trials can no longer be described as a “gold standard” for assessment of pharmacotherapy aimed at improving symptom endpoints, e.g., antianginals. They are now merely a minimum standard for any symptom efficacy data to be taken seriously.

**Fig. 1:**
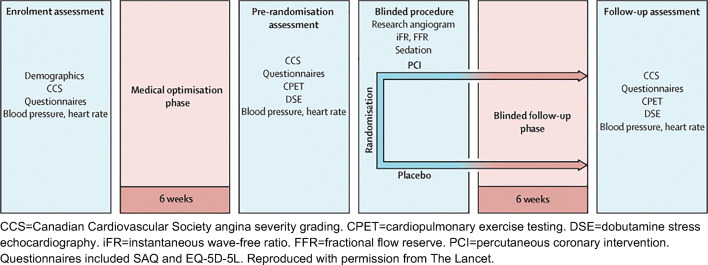
ORBITA study design. CCS = Canadian Cardiovascular Society angina severity grading, CPET = cardiopulmonary exercise testing, DSE = dobutamine stress echocardiography, iFR = instantaneous wave-free ratio, FFR = fractional flow reserve, PCI = percutaneous coronary intervention. Questionnaires included SAQ and EQ-5D-5L. Reproduced with permission from The Lancet

Unfortunately, this minimum standard for drugs is considered a maximum standard for device intervention, and sometimes the term “gold standard” is applied as though it were an unattainable academic icing on the cake. Where clinical adoption of an intervention would carry any short or long-term risk, placebo-controlled trials should be considered the entry standard for assessment of symptom and QoL endpoints. Fig 1

## Key Trials of Revascularization for Chronic Stable Angina

Several unblinded trials have been performed assessment PCI or revascularization in chronic stable angina (Table 2). For the majority, the primary endpoint was death and MI; however, since these endpoints rarely showed favorable results for revascularization, interventional cardiologists inevitably refocused the conversation about these trials on secondary endpoints of symptoms and QoL. [23]

**Table 2:** Trials of PCI for angina assessing quality of life

Trial	N	QoL assessment tool	Blinded	Result
ACME	182	QoL questionnaire with physical and psychological items	No	Improvement in QoL scores in the PCI group compared with medical therapy
ACME2	328	QoL questionnaire with physical and psychological items	No	No difference between PCI and medical therapy groups in QoL scores at 6 months
RITA-2	1018	SF-36	No	Improved QoL in PCI group but not sustained at 3 years
MASS	214	Angina freedom	No	Greater angina freedom in CABG and PCI groups compared to medical therapy
MASS2	611	SF-36	No	Improved QoL in CABG and PCI groups compared to medical therapy
COURAGE	2287	SAQ	No	Improved health status in PCI group but benefit disappeared by 36 months
FAME2	888	EQ-5D	No	Improved QoL in PCI group
ISCHEMIA	4617	SAQ summary score	No	Greater improvement in invasive strategy group compared with conservative strategy group
ORBITA	200	SAQ, EQ-5D-5L	Yes	No difference between PCI and placebo groups in QoL scores at 6 weeks

*CABG c*oronary artery bypass grafting, *EQ-5D* 5-dimension EuroQoL, *PCI p*ercutaneous coronary intervention, *QoL* quality of life, *SAQ* Seattle Angina Questionnaire, *SF-36* 36 item short form survey

In 1992, the Angioplasty Compared to MEdicine (ACME) trial was the first study to comprehensively QoL in a stable angina cohort [24]. It randomized 212 patients with single-vessel disease to percutaneous balloon angioplasty (POBA) or medical therapy and found dramatic improvements in symptoms and exercise time with POBA compared to medical therapy. An unblinded 2.1 ± 3.1-min improvement in exercise tolerance was seen in the POBA group compared to 0.5 ± 2.2 min in the medical therapy group. A follow-up publication reported self-assessed QoL in 182 of the participants and completed at baseline and at 6-months follow-up [25]. Both physical and psychological QoL domains were assessed. The McMaster Health Index Questionnaire was used to assess the physical domain. There was greater improvement in both psychological and physical wellbeing in the POBA arm.

This symptomatic benefit was not replicated in the ACME2 trial which looked at double-vessel disease [26]. It showed no difference in 6-month exercise time improvement in patients with single-vessel disease who were randomized to PCI or medical therapy (PCI 1.2 min versus OMT 1.3 min, *p* = 0.89). There was also no difference in self-assessed QoL.

In 1995, the Medicine, Angioplasty or Surgery Study (MASS) examined 214 patients with chronic stable angina and single proximal left anterior descending artery stenosis, who were randomized to medical therapy, PCI or CABG [27]. Long-term secondary outcomes included angina functional class at last follow-up and employment status at 2 years. The investigators found that intervention with either revascularization or PCI achieved significantly higher rates of angina-free status at 3 years (98% and 82% of patients respectively) compared to the medical therapy arm (32%). There was no difference in employment status at 2 years.

The second Randomised Intervention Treatment of Angina trial (RITA-2) showed that PCI significantly increased mortality, the primary endpoint [28]. Understandably, the focus shifted to a greater improvement in SF-36 scores in the PCI arm compared to the medical therapy only arm at 1 year. Interestingly, the improvement was not sustained for 3 years [29]. A similar pattern was seen with exercise time with a superior improvement of 35 s at 3 months in the PCI arm, reducing to 25 s at 1 year [28].

MASS 2 compared medical therapy alone versus PCI or CABG with medical therapy. The CCS scale was used to assess angina with 79% having class 2 or 3 angina at baseline. At 1-year follow-up, 36% of the medical therapy alone, 52% of the PCI arm, and 59% of the CABG arm were angina-free. QoL assessment using the SF-36 tool at baseline, 6 months, and 1 year showed higher QoL in the PCI and CABG arms compared to the medical therapy only arm [30].

The COURAGE trial randomized 2287 patients to PCI and optimal medical therapy or optimal medical therapy alone. There were improvements in all the SAQ domains in the PCI arm compared to the control group [31]. Patients in the PCI arm were more likely to become angina free.

A follow-up analysis of the landmark Fractional Flow Reserve–Guided PCI versus Medical Therapy in Stable Coronary Disease (FAME-2) study examined QoL in 888 of the 1220 originally enrolled patients using EQ-5D questionnaire. Patients in the PCI group had significantly improved QOL up to 2 years post-procedure [32].

The ISCHEMIA trial randomized patients with moderate-to-severe ischemia to an invasive versus conservative approach and showed a significant improvement in angina [33] in the invasive group at median follow-up of 3.2 years. While unblinded trials have shown significant improvements in symptoms and QoL with invasive approaches of PCI or CABG, it is important to note that these reported effect sizes are not bias-resistant, and the impact of placebo remains unknown.

## Insights from ORBITA

While placebo control is mandatory for pharmacotherapy, it is rarely used for interventions [34]. Various barriers to placebo-controlled trials are cited such as a concern that it might be unethical to perform, in a trial volunteer, a placebo procedure. Oddly, there is rarely a mention of the converse concern that an ineffective procedure might become widely adopted clinically and therefore harm a great many more people who did not volunteer for a placebo procedure.

The Objective Randomised Blinded Investigation with Optimal Medical Therapy of Angioplasty in Stable Angina (ORBITA) trial was the first placebo-controlled study of PCI for chronic stable angina [11]. It recruited patients with symptoms of angina or equivalent with angiographically severe single-vessel disease at 5 UK sites. They underwent a 6-week medical optimization phase consisting of uptitration of guideline-directed antianginal medication, aiming for guideline-directed medical therapy, by means of telephone consultation with a consultant cardiologist. Prior to randomization patients were assessed with cardiopulmonary exercise testing, dobutamine stress echo (DSE), CCS classification, SAQ, and EQ-5D-5L. Patients were then randomized to undergo PCI or a placebo procedure after coronary angiography, following FFR and iFR measurements with the interventional consultant cardiologist blinded to these findings. Patients then completed a 6-week blinded follow-up phase which ended with follow-up assessment with repeat assessment of all pre-randomization tests.

The hypothesis was that in patients with angina and severe single-vessel coronary artery disease on optimum medical therapy, PCI would result in a greater improvement in treadmill exercise time, symptoms, and QoL than a placebo procedure. The results were surprising despite 94% of patients having evidence of ischemia on one or more non-invasive or invasive functional tests.

ORBITA showed a smaller than expected and not statistically significant placebo-controlled effect size of PCI on change in exercise time. It also showed no significant improvement beyond placebo in symptoms as assessed by CCS and SAQ or QoL as assessed by EQ-5D-5L. This was despite a clear improvement in DSE (improvement of peak wall motion index score) in the PCI arm versus placebo. The only symptom endpoint where placebo-controlled efficacy of PCI was seen was in the non-prespecified analysis of SAQ freedom from angina in which 1 in 5 more patients in the PCI arm were free from angina at follow-up than in the placebo arm [35].

ORBITA has taught us that it is safe and feasible to perform placebo-controlled trials of PCI. It has also shown the surprisingly small effect size that is seen when the placebo component is subtracted from the overall effect size to calculate the true physical effect of an intervention for symptoms and QoL.

## ORBITA-2

The second blinded placebo-controlled trial of PCI for angina (ORBITA-2) is currently in the recruitment phase [36]. This trial aims to build upon the results of ORBITA to apply similar trial methodology to a wider range of patients with chronic stable angina. ORBITA-2 will recruit 400 patients with symptoms of angina, single- or multi-vessel disease, and evidence of ischemia in a blinded trial with a 12-week follow-up period. At enrollment, regular antianginal medications are stopped in order to assess the true effect of PCI versus placebo in patients on real-world medical therapy. If symptoms become intolerable, antianginal medication is introduced. At randomization, any regular antianginals are again stopped and are restarted during the blinded follow-up phase using a protocolized approach if symptoms are intolerable.

ORBITA-2 will provide an estimate of the placebo-controlled effect of PCI on a patient-reported primary outcome measure that has been co-designed with patients. Secondary endpoints of CCS class, SAQ, EQ-5D-5L, and MacNew questionnaires will also be reported.

## Other Blinded Trials

There are only a handful of blinded (and therefore more bias-resistant) trials of interventional procedures for angina.

Internal mammary ligation was initially thought to improve angina from unblinded experiences in the 1950s. Only when the double-blind trial exposed it as ineffective was the procedure abandoned [37].

Similarly, percutaneous transmyocardial laser revascularization was widely considered to be a both logical and effective treatment for angina in the 1990s. When the placebo-controlled trial showed no effect on exercise time, CCS class, or SAQ, it too was abandoned [38].

The Coronary Sinus Reducer for Treatment of Refractory Angina (COSIRA) trial randomized 104 patients with refractory angina and not suitable for revascularization, to receive the coronary sinus reducer device or placebo [39]. The primary endpoint was a change in CCS class with secondary endpoints of SAQ QoL and treadmill exercise time. The coronary sinus reducer was associated with improvement in both physician-assessed CCS symptoms and QoL. However, there was no difference in treadmill exercise time between the intervention and placebo-groups.

The SHam-controlled INtErvention to improve QoL in Chronic Total Occlusions (SHINE-CTO) trial is underway, randomizing patients with angina and a chronic total occlusion to PCI or placebo [40]. The primary endpoint is the SAQ summary acore.

One argument that is frequently made is that blinding is expensive and practically difficult. However, these examples show that it is not only possible but actually necessary.

## Conclusion

Conventional teaching, clinical experience, and unblinded trials tell us that revascularization improves QoL but this was not supported by the only blinded trial of PCI, ORBITA. Another blinded trial, ORBITA-2, is underway and will add to the blinded data in the field. The difference in effect size when comparing unblinded to blinded data shows us that placebo-controlled trials are required to reduce bias in QoL assessment and should become the minimum standard for trials of any intervention with symptom or QoL endpoints in order to evaluate the true physical effect of the treatment beyond placebo.

## Data Availability

Data will be made available on request.

## Declarations

This article does not contain any studies with human participants performed by any of the authors. In ORBITA, informed consent was obtained from all individual participants included in the study.
